# Depression Dimensions: Integrating Clinical Signs and Symptoms from the Perspectives of Clinicians and Patients

**DOI:** 10.1371/journal.pone.0136037

**Published:** 2015-08-27

**Authors:** Edgar Arrua Vares, Giovanni Abrahão Salum, Lucas Spanemberg, Marco Antônio Caldieraro, Marcelo P. Fleck

**Affiliations:** 1 Department of Psychiatry, Hospital de Clínicas de Porto Alegre, Universidade Federal do Rio Grande do Sul, Porto Alegre, Brazil; 2 Department of Psychiatry, Pontifícia Universidade Católica do Rio Grande do Sul, Porto Alegre, Brazil; Federal University of Rio de Janeiro, BRAZIL

## Abstract

**Background:**

Several studies have recognized that depression is a multidimensional construct, although the scales that are currently available have been shown to be limited in terms of the ability to investigate the multidimensionality of depression. The objective of this study is to integrate information from instruments that measure depression from different perspectives–a self-report symptomatic scale, a clinician-rated scale, and a clinician-rated scale of depressive signs–in order to investigate the multiple dimensions underlying the depressive construct.

**Methods:**

A sample of 399 patients from a mood disorders outpatient unit was investigated with the Beck Depression Inventory (BDI), the Hamilton Depression Rating Scale (HDRS), and the Core Assessment of Psychomotor Change (CORE). Exploratory Factor Analysis (EFA) and Confirmatory Factor Analysis (CFA) were used to investigate underlying dimensions of depression, including item level analysis with factor loadings and item thresholds.

**Results:**

A solution of six depression dimensions has shown good-fit to the data, with no cross-loading items, and good interpretability. Item-level analysis revealed that the multidimensional depressive construct might be organized into a continuum of severity in the following ascending order: sexual, cognitive, insomnia, appetite, non-interactiveness/motor retardation, and agitation.

**Conclusion:**

An integration of both signs and symptoms, as well as the perspectives of clinicians and patients, might be a good clinical and research alternative for the investigation of multidimensional issues within the depressive syndrome. As predicted by theoretical models of depression, the melancholic aspects of depression (non-interactiveness/motor retardation and agitation) lie at the severe end of the depressive continuum.

## Introduction

Major depressive disorder (MDD) has increasingly been considered a multidimensional construct [1–3]. There is some preliminary evidence showing that different MDD dimensions might originate from different etiologies [4], and that some particular symptom dimensions might predict poor outcomes with antidepressant treatment [3]. Therefore, methods to properly assess the multidimensionality of MDD could assist the exploration of etiological investigations and lead to advances in treatment choice. Although there are several studies investigating the dimensions underlying MDD using a variety of symptomatic scales and statistical methods [2, 5–7], evidence for the number and structure of depressive symptoms is still a matter of debate [5].

Previous evidence is limited in a number of important ways. *First*, depression-rating scales are generally constructed based on theoretical grounds and designed for particular aims, such as to evaluate treatment response in clinical trials. Therefore, it is unlikely that a single rating scale would be able to capture the detailed phenomenological heterogeneity of MDD [5]. It has been recently suggested that individual symptomatic scales might have insufficient item coverage to account for the multidimensionality of depression. For example, Brouwer et al. [8], while studying the Beck Depression Inventory (BDI), found that the total scale score variation reflected some multidimensionality, but not enough to justify the scoring of subscales. Therefore, although theoretical and empirical views of MDD recognize the importance of multidimensionality for the depressive syndrome, available symptomatic scales might be limited in their ability to provide a proper multidimensional assessment because of their insufficient item content.

*Second*, in many clinical and research situations scales are often used in populations different than the ones they were designed for. The Hamilton Depression Rating Scale (HRSD) has been shown to be composed of sub-dimensions [7]. However, as it is a scale developed for assessing the severity of depression in clinically depressed patients, it may lack items that covers symptoms more commonly encountered in less severe forms of depression [9].

*Third*, there is no clearly established consensus in regards to which would be the best way of evaluating the multidimensionality of depression. One might hypothesize that different depression dimensions might benefit from different perspectives. For example, as noted by Uher et al. [10], while some signs might be better evaluated by clinical observation (e.g., psychomotor agitation), some symptoms might be better assessed by clinical interview (e.g., guilt), and finally other symptoms may be more easily assessed by self-report because of their intimate nature (e.g., sexual symptoms). Therefore, integration of clinically evaluated signs (frequently ignored in the literature) and symptoms with both patient and clinician reported symptoms might allow for a better evaluation of different aspects of depression, which cannot be reached by way of each specific perspective individually. There is evidence [11, 12] that self-reported and clinician-rated outcomes are not equivalent, and that each of these two ratings may provide distinctive information that may be relevant to clinical prognosis. The absence of a gold standard for the assessment of depression led some authors [11, 13, 14] to suggest that both of these assessment modalities should be combined, since each of them may provide unique non-redundant information that complements the other in predicting treatment outcomes. A large meta-analysis examining the effect sizes of psychotherapy for adult depression included only studies in which both a self-reported and a clinician-rated instrument were used [15]. Results showed that clinician-rated instruments presented significant higher effect size than self-reported instruments from the same studies [15].

*Finally*, the total scores of the rating scales conceal multidimensionality. Current practices rely solely on summing up scale items, in accordance with Classical Test Theory methods, which assume that all items from a scale measure the latent construct with equal accuracy (parallelism). This does not seem to be a realistic assumption. In fact, it is improbable that each item from depression scales will discriminate depression severity equally well in every person from a given population [16]. As a practical example of this situation, one depressed patient presenting suicidal thoughts and another patient presenting a decrease in appetite will score the same, with no room to differentiate these ratings. Therefore, analysis strategies that aim to investigate differential item contributions to the multidimensional aspects of depression might provide valuable information to clinicians and researchers when rating the severity of depression.

These limitations are addressed in a large sample of outpatients with MDD. We integrate information from three instruments that target different aspects of the depressive syndrome: the 21-item Beck Depression Inventory (BDI), a self-reported patient rated scale; the 17-item Hamilton Depression Rating Scale (HDRS), a clinician-rated symptom and sign scale; and the Core Assessment of Psychomotor Change (CORE), which is a clinician-rated inventory for depression signs. The BDI was developed based on records of statements made by individuals with depressive disorders during psychotherapeutic sessions [17], which results in a large proportion of its items being focused on the cognitive symptoms of depression. The HDRS was developed and validated for use with psychiatric inpatients presenting unipolar and bipolar affective disorders [18], which results in many of its items assessing symptoms more commonly encountered in more severely affected depressive patients; furthermore, the HDRS is a scale that seems to cover a significant proportion of the depressive phenomena. Therefore, by choosing these two instruments, in addition to one being self-reported and the other clinician-rated, we intended to integrate these two different clinical perspectives: instruments assessing different profiles of depressive symptoms. A third and significant aspect, not sufficiently well appraised by both of these scales, is psychomotor disturbance. The HDRS has only two items assessing psychomotor disturbance by direct observation, and the BDI has none. It would be natural to consider that an instrument that is based on direct observation would better access signs of psychomotor disturbance. The CORE measure meets this goal, since it is an operationalized observer-rated instrument evaluating cognitive processing disorders (non-interactiveness), agitation, and motor retardation [19]. Another important asset of the CORE instrument is the way it was developed: following strictly scientific and cogent ideas about melancholia [20], and not as an instrument merely designed for assessing the effectiveness of antidepressant medications. First, an Exploratory Factor Analysis (EFA) is performed in order to obtain a fine-grained description of the three scales covariance structure (the latent dimensionality of the three instruments comprised), and then, differential items contributions are estimated using Confirmatory Factor Analysis (CFA).

The advantages of combining clinician and self-rated signs and symptoms of depression in a dimensional analysis are: 1) having more items in total–what in turn will provide more items per dimension, allowing more proper scoring of these dimensions; 2) integrating different perspectives of measurement–putatively, self-rated scales more sensitive to subjective symptoms, and clinician-rated scales more sensitive to objective signs of depression; 3) a more comprehensive assessment than it would be possible by means of each instrument separately.

This is mostly an exploratory work. Nonetheless, we would expect probably finding a psychomotor dimension (with items from the CORE and HDRS), a cognitive dimension (with items mostly from the BDI), and a mood dimension (with items from the BDI and HDRS).

## Materials and Methods

### Sample selection

Patients from a general hospital tertiary outpatient depression unit were invited to participate in the study. The inclusion criteria consisted of a primary diagnosis of major depressive disorder (MDD), as defined by the DSM-IV and ICD-10, and assessed by the Mini International Neuropsychiatric Interview Plus (M.I.N.I. Plus), Brazilian version [21]. The M.I.N.I. Plus is a more detailed version than its original one, that helps mainly with the diagnosis of psychotic and mood DSM-IV disorders, and explores other clinical diagnoses not covered in the shorter version. The exclusion criteria included a history of manic or hypomanic episodes, a neurological disorder that could hamper the assessment of psychomotor disturbance, and not being able to understand the self-rated instruments. Five hundred eighty patients referred from the primary care system from July 2009 to June 2013 with a presumptive diagnosis of unipolar major depressive disorder were invited to participate in the study, and 399 patients accepted participation and met the inclusion criteria. Of these 580 patients, 22 did not accept to participate, 40 did not meet the criteria for major depressive episode according to M.I.N.I. Plus, 20 did not complete the diagnostic interview, 32 were not able to fill out the self-reported questionnaires, and 67 had a history of manic or hypomanic episodes. This research was approved by the Ethic Committee of the Hospital de Clínicas de Porto Alegre (HCPA). All patients provided a written informed consent form, which had been previously approved by the institutional review board from the Hospital de Clínicas de Porto Alegre.

### Measurement instruments

The Brazilian Portuguese version [22] of the Beck Depression Inventory (BDI) [17] was utilized. The BDI is a 21-item self-reported patient rated scale evaluating symptoms of depression. In addition, it is among the most extensively used self-rated instruments in clinical and research settings [23]. It has been widely translated into many languages, and its Brazilian Portuguese version has shown psychometric properties comparable to its English version, with a Cronbach’s alpha of 0.88 for depressed patients and 0.81 for controls [22]. The 21 items, each scored on a scale of 0 to 3, address the following issues: 1) sadness, 2) future pessimism, 3) lack of enjoyment, 5) guilt, 6) feelings of being punished, 7) disappointment with oneself, 8) self-blame, 9) suicidal thoughts, 10) crying, 11) irritability, 12) interest in people, 13) making decisions, 14) appearance, 15) work, 16) sleep, 17) tiredness, 18) appetite, 19) weight loss, 20) health anxiety, and 21) interest in sex.

The Hamilton Depression Rating Scale (HDRS) [24] version used in the present study is a 17-item clinician rated scale evaluating signs and symptoms of depression [18]. It is one of the most utilized instruments worldwide for the assessment of the severity of the depressive syndrome [25]. The HDRS was developed in the late 1950s with the objective of evaluating the effectiveness of antidepressant treatment, although it has come to be considered by many to be the gold standard for the measurement of depression. In spite of this, more current evidence has suggested that its psychometric properties and validity present some important limitations, which are mainly related to its lack of unidimensionality, having items that do not cover the full spectrum of the depressive syndrome, and items loosely related to the DSM concept of depression [9, 25]. The 17 items, with their range of responses, address the following issues: 1) depressed mood (0–4), 2) feelings of guilt (0–4), 3) suicide (0–4), 4) early insomnia (0–2), 5) middle insomnia (0–2), 6) late insomnia (0–2), 7) work and activities (0–4), 8) psychomotor retardation (0–4), 9) agitation (0–4), 10) psychological anxiety (0–4), 11) somatic anxiety (0–4), 12) gastrointestinal somatic symptoms (0–2), 13) general somatic symptoms (0–2), 14) genital symptoms (0–2), 15) hypochondriasis (0–4), 16) weight loss (0–2), and 17) insight (0–2). The ratings covered the 1-week period prior to the interview.

The Core Assessment of Psychomotor Change (CORE) [20] is an 18-item clinician rated scale evaluating psychomotor signs of depression. The utilized version was culturally adapted and translated into Brazilian Portuguese, and then applied by our group in accordance with the guidelines from the International Society for Pharmacoeconomics and Outcomes Research [26]. The process utilized has been described elsewhere using another scale as an example [27]. It is intended to be used when a primary diagnosis of major depression has been made, and to differentiate the melancholic from the non-melancholic subtype [20]. Ratings are based on subjects’ observed behavior during the interview, and not on subjective feelings [20]. Furthermore, being that the instrument rates subtle observed behavioral differences, clinical experience with depressive and other psychiatric and medical patients is necessary. Signs should first be judged to be categorically present or absent (quality), and then, if present, to be graded in severity (quantity). A score of 0 indicates that the sign is absent or trivial, while scores of 1 to 3 indicate definite presence with increasing severity. This is in line with the author’s conceptualization of melancholic depression, as a categorical-dimensional disorder [19]. The items on the standard CORE rating form are intentionally presented in random order. It is assumed that there is a main factor underpinning the CORE (non-interactiveness), which splits into retardation and agitation factors. Six items represent the non-interactiveness factor in the CORE measure, 7 items represent the retardation factor, and 5 items represent the agitation factor. Finally, the CORE comprises 18 items, each scored 0–3, and divided into three subscales representing the three above-mentioned factors. The non-interactiveness items are: 1) non-interactiveness (item 1), 2) non-reactivity (item 4), 3) inattentiveness (item 8), 4) poverty of associations (item 12), impaired spontaneity of talk (item 16), and length of verbal responses (item 7). The retardation items are: 1) slowed movement (item 13), 2) facial immobility (item 2), 3) body immobility (item 10), 4) postural slumping (item 3), 5) delay in motor activity (item 15), 6) delay in responding verbally (item 6), and 7) slowing of speech rate (item 17). The agitation items are: 1) facial apprehension (item 5), 2) facial agitation (item 9), 3) motor agitation (item 11), 4) stereotyped movement (item 18), and 14) verbal stereotypy (item 14).

### Diagnostic procedures

Three psychiatrists (EAV, MAC and LS), experienced in the evaluation and treatment of depression, conducted the clinical assessments. All psychiatrists had 6 years of medical school and at least 3 years of psychiatric training, with a minimum of six months of training with the assessment instruments. The psychiatrists were trained with an informational video before using the CORE. With the aim of increasing inter-rater reliability, the three psychiatrists together performed the first six months of assessments. Medical students delivered the self-reported questionnaires, and when patients were not able to respond them by themselves due to vision disturbances or illiteracy, the medical students were instructed to read them aloud and explain any misunderstood item (assisted application). The medical students were instructed not to interpret the items for the patients.

### Statistical Analysis

The aim of factor analysis is to determine the number and nature of latent variables (factors) that are responsible for the variation and covariation among a series of observed measurements (indicators) [28]. Since no previous studies have investigated dimensions of depression using both patient and clinician-rated scales using symptoms and signs of depression, an Exploratory Factor Analysis (EFA) was performed. Being that we were dealing with categorical variables, we used the robust weighted least squares (WLSMV) as an estimator for both the EFA and CFA [28], implemented with Mplus 7.0 [29].

For the EFA, the selection of the number of factors took into account the scree plot of eigenvalues, items cross-loadings, statistical indices, and theoretical interpretability according to models proposed by Uher et al [10] and Parker et al [20]. The statistical indices taken into account were: chi-square, comparative fit index (CFI), Tucker-Lewis index (TLI), root mean square error of approximation (RMSEA), and standardized root mean square residual (SRMR). To demonstrate good fit to the data, research suggests that an estimated model should have SRMR values close to .08 or below, RMSEA values close to .06 or below, and CFI and TLI values close to .95 or greater [30]. With the objective of fostering interpretability (maximize high loadings, minimize low loadings) the EFA was performed using the Geomin rotation. Stevens [31] recommends interpreting only factor loadings with an absolute value greater than 0.4, which would explain around 16% of the variance in the variable; thus, only items with factor loadings over 0.4 were considered. Theoretical and clinical interpretability, and significance of the factors, were likewise taken into account for selecting the number of factors.

After selection of the best model, a Confirmatory Factor Analysis (CFA) was performed in order to investigate the adjustment of the model to our sample, along with model-based item factor loadings and thresholds. The CFA model was fitted to polychoric correlations using as estimator and goodness-of-fit indices the same parameters mentioned above (for the EFA), except for using the WRMR instead of the SRMR, which should be near or below .9 [29]. CFA can also be used to investigate the latent dimensionality of categorical outcomes. Factor loading indicates how well the item reflects the underlying dimension and how well the item performs in terms of discriminating subjects within the latent trait. The factor loading also informs the relative contribution that each item makes for the latent variable, which means that the higher the factor loading, the stronger the association between them will be. Threshold parameters reflect the standardized level of depression severity at which subsequent response options become more probable than the previous option. CFA with categorical indicators is equivalent to Item Response Theory (IRT), with factor loadings analogous to item discrimination parameters, and item thresholds to item difficulty parameters or item location parameters.

According to Brown [28], a common sequence in scale development and construct validation is to conduct CFA as the next step after latent structure has been explored using EFA. Still, the researcher frequently encounters a poor-fitting CFA solution because of the potential sources of misfit that are not present in EFA (e.g., indicator cross-loadings and residual covariances usually fixed to zero). Therefore, because of the restrictions commonly imposed on the factor solution when moving from the EFA to the CFA, a deterioration of the goodness-of-fit indices can usually be expected. Yet, the procedure of EFA within the CFA framework can be a useful precursor to CFA that allows the researcher to explore measurement structures more fully before moving into a confirmatory framework–this approach represents an intermediate step between EFA and CFA that provides substantial information important in the development of realistic confirmatory solutions [28].

## Results

The final sample consisted of 399 patients with unipolar depression. Socio-demographic and clinical characteristics of the sample are presented in Table 1. The BDI and HDRS mean scores indicated severe depression, and the CORE mean score indicated non-melancholic depression; these mean scores relates to the total sample.

**Table 1:** Socio-demographic and clinical characteristics of the sample (n = 399).

Age, mean (+-SD)	49.84 (11.13)
Female gender, n (%)	350 (87.6%)
Years of education, mean (+-SD)	7.37 (3.65)
Ethnicity, n (%)	
White	303 (75.9%)
Others	92 (24.1%)
BDI, mean (+-SD)	33.68 (10.25)
HDRS, mean (+-SD)	20.37 (5.40)
CORE, mean (+-SD)	5.19 (5.07)

Legend: BDI, 21-item Beck Depression Inventory; HDRS, 17-item Hamilton Depression Rating Scale; CORE, Core Assessment of Psychomotor change.

### Exploratory Factor Analysis (EFA)

**Fig 1:**
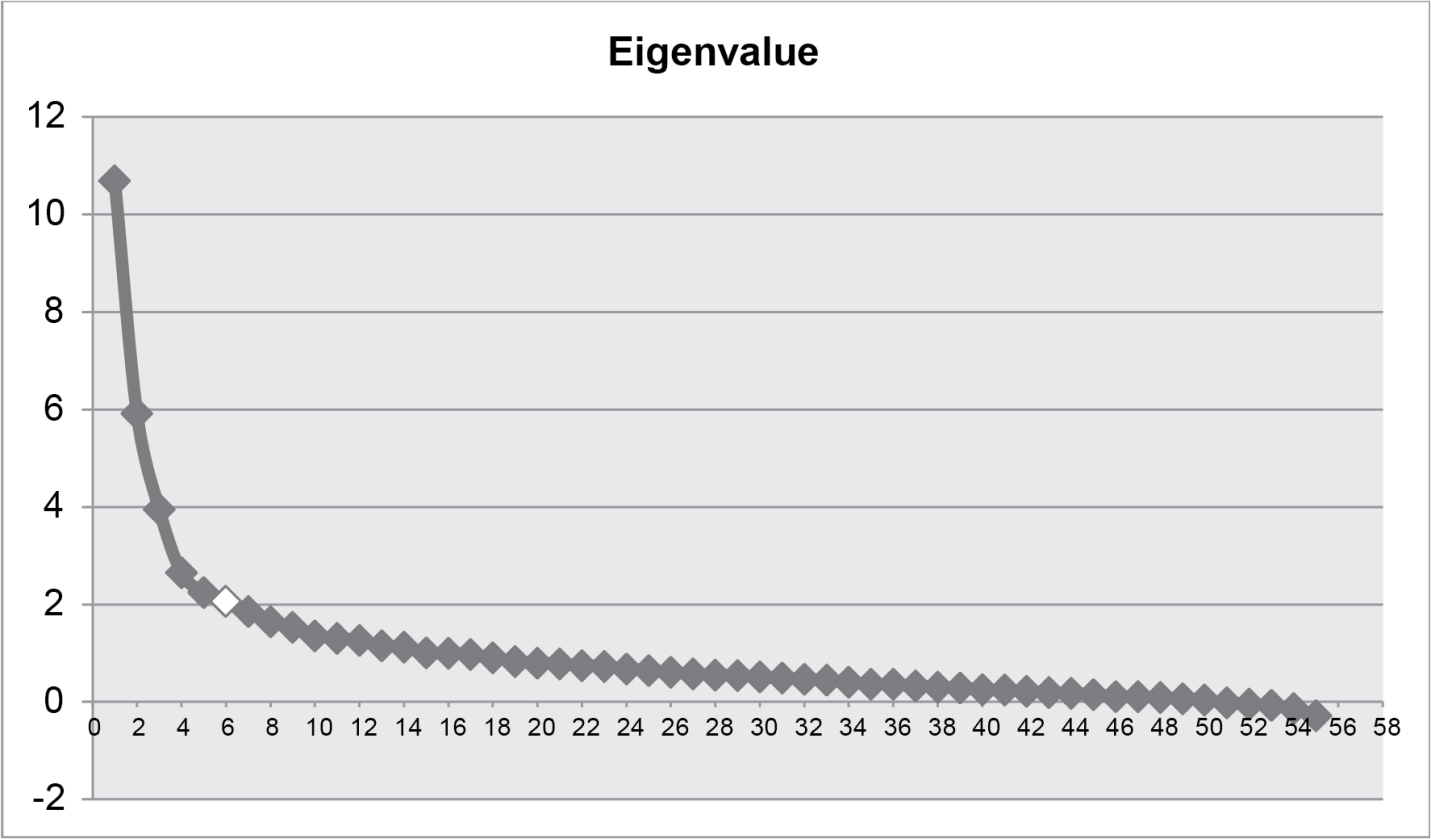
Exploratory Factor Analysis (EFA) *eigenvalues*. Legend: Horizontal axis: number of factors; vertical axis: factor *eigenvalues*. The six-factor solution provided the most parsimonious and interpretable description.

The result of the scree plot (see Fig 1) with the 56 items of the composite of the three scales (BDI, HDRS and CORE) favored a solution of six factors, with the following goodness-of-fit indices for this solution: a chi-square of 1829.205, a CFI of 0.931, a TLI of 0.912, a RMSEA of 0.035, and a SRMR of 0.074. Although it is not indicative of a perfect fit, these indices can be considered to be performing reasonably well for such a complex construct. With the objective of attaining a pragmatic equilibrium between model fit and applicability, the solution of six factors was favored in relation to others with more factors. Whereby, despite presenting better goodness-of-fit indices, the clinical interpretability was not as consistent as the one with six factors. In addition, solutions with 7–10 factors presented a high number of cross-loading and dimensions that were not clearly interpretable. Just one item presented a cross-loading: item CORE 9 (facial agitation) loaded on both factors 1 and 3 (but since this item loaded much better in one factor than in the other, this item was retained). The item CORE 18 was excluded because of its reduced frequency (98.5% of ratings = 0). A total of 13 items were later discarded because they did not load on any factor. Solutions from 2 to 10 factors were tested, and they are presented inS1 File.

The first factor was an insomnia factor, with items from HDRS and BDI; the second factor was a motor retardation/non-interactiveness factor, essentially with items from the CORE; the third factor was an agitation factor, again with most items from the CORE, and two more items from the HDRS; the fourth factor was a cognitive factor, basically with items from the BDI; the fifth and sixth factors were an appetite and a sexual factor, respectively, with a mix of items from HDRS and BDI. The six-factor solution is presented in Table 2.

**Table 2:** Exploratory Factor Analysis six-factor solution.

Scale / Item	Item dimension (Uher / Parker)	Item content	Factor loadings					
			Factor 1	Factor 2	Factor 3	Factor 4	Factor 5	Factor 6
HAM6	Sleep	insomnia late	**.637**	-.011	.014	.096	.169	-.047
HAM5	Sleep	insomnia middle	**.593**	.029	-.014	.155	.075	-.003
BDIQ16	Sleep	insomnia	**.440**	.015	-.085	.221	.088	.060
CORE17	Retardation	slowing of speech	.033	**.915**	-.148	-.134	.077	-.102
HAM8	none	psychomotor retardation	-.008	**.905**	-.101	-.109	.105	-.078
CORE6	Retardation	delayed verbal responses	.080	**.897**	.039	-.097	-.075	.031
CORE16	Non-interactiveness	talk impaired spontaneity	-.033	**.856**	.013	.114	-.058	-.091
CORE7	Non-interactiveness	shortened verbal responses	.006	**.851**	.060	.009	-.074	-.143
CORE13	Retardation	slowed movement	.125	**.795**	-.219	-.097	.089	.098
CORE12	Non-interactiveness	poverty of associations aaaasassociations	-.041	**.786**	.075	-.008	-.130	-.084
CORE2	Retardation	facial immobility	-.097	**.769**	-.037	.072	.113	.035
CORE1	Non-interactiveness	non-interactiveness	-.152	**.769**	-.037	.067	-.068	.123
CORE10	Retardation	bodily immobility	-.012	**.729**	-.079	.124	.035	-.144
CORE15	Retardation	delayed motor activity	.044	**.725**	-.111	.008	-.139	.237
CORE4	Non-interactiveness	non-reactivity	-.014	**.688**	.229	.144	.089	.029
CORE8	Non-interactiveness	inattentiveness	-.023	**.534**	.276	-.081	-.187	.157
CORE9	Agitation	facial agitation	**.494**	-.052	**.776**	-.047	-.072	-.030
CORE11	Agitation	motor agitation	-.015	-.095	**.887**	.077	.064	-.244
HAM9	Anxiety	agitation	-.076	-.107	**.798**	-.010	.017	-.214
CORE5	Agitation	facial apprehension	.337	.207	**.705**	-.080	.031	.021
CORE14	Agitation	verbal stereotypy	-.021	.058	**.624**	.099	-.166	.164
HAM10	Anxiety	anxiety psychological	.301	.076	**.564**	-.057	.021	.049
BDIQ5	Pessimism	feelings of guilt	.078	-.020	-.115	**.825**	.012	-.326
BDIQ3	Pessimism	feelings of failure	.045	.022	.030	**.724**	-.054	.028
BDIQ7	Pessimism	disappoint in myself	.031	.093	.082	**.659**	.005	-.057
BDIQ6	Pessimism	feelings of punishment	.159	-.035	.017	**.621**	.003	-.160
BDIQ8	Pessimism	critical of myself	.187	.006	-.010	**.591**	-.035	-.061
BDIQ9	Pessimism	suicidal thoughts	-.067	.068	.138	**.585**	.158	.018
BDIQ12	Interest-activity	lost interest in people	-.067	-.068	-.072	**.571**	.119	.125
BDIQ13	Interest-activity	difficulties in decisions	.049	.080	.033	**.565**	.025	.017
BDIQ1	none	feelings of sadness	-.026	.043	.047	**.561**	.040	.142
BDIQ2	Pessimism	hopelesness	-.095	-.063	-.097	**.540**	-.038	.262
BDIQ4	Interest-activity	anhedonia	-.026	-.150	-.060	**.502**	-.028	.089
HAM3	Mood	suicide	-.094	.141	.192	**.486**	.162	-.001
BDIQ14	Pessimism	unatractiveness (feel.)	.136	-.013	.078	**.480**	-.123	.068
HAM2	Pessimism	feelings of guilt	.105	-.002	.002	**.416**	-.017	-.313
HAM16	Appetite	weight loss	.033	-.010	-.023	-.085	**.902**	-.001
BDIQ19	Appetite	weight loss	.073	-.031	.009	.014	**.727**	-.071
HAM12	Appetite	GI symptoms	.039	.146	.038	.030	**.660**	.116
BDIQ18	Appetite	worse appetite	-.042	.031	.027	.176	**.589**	.189
HAM14	Interest-activity	genital symptoms	.173	-.164	.070	.009	.040	**.663**
BDIQ21	Interest-activity	loss of libido	.039	-.184	-.035	.114	.093	**.636**
BDIQ15	Interest-activity	working difficulties	-.016	.217	.027	.275	.046	.331
BDIQ17	Interest-activity	tiredness	-.051	.025	.016	.274	.000	.300
HAM13	Interest-activity	somatic symptoms	.110	.144	-.107	.084	-.030	.283
BDIQ10	none	crying	-.069	.191	-.027	.241	-.005	.223
BDIQ11	none	irritation	-.020	-.035	.008	.253	.046	.174
BDIQ20	none	worry physical	.215	.107	-.029	.246	-.034	.173
HAM11	Anxiety	anxiety somatic	.145	.032	.107	-.062	.053	.138
HAM15	Anxiety	hypochondriasis	.379	.035	.001	.044	-.134	.135
HAM1	Mood	depressed mood	.109	.395	.306	.095	.063	.133
HAM7	Interest-activity	work and activities	-.214	.333	.048	.207	.148	.064
HAM4	Sleep	insomnia early	.380	-.053	-.011	.120	-.045	.057
HAM17	none	insight	-.094	.078	.013	-.004	-.026	-.038
CORE3	Retardation	postural slumping	.122	.348	.276	.097	.026	-.104

Legend: HAM, 17-item Hamilton Depression Rating Scale; BDI, 21-item Beck Depression Inventory; CORE, Core Assessment of Psychomotor change. The items that did not enter the models by Uher and Parker are coded in the “none” item dimension category.

### Confirmatory Factor Analysis (CFA)

The CFA was performed with the resulting 44 items from the EFA six-factor solution. The goodness-of-fit indices are as follows: a chi-square of 1480.451, a CFI of 0.909, a TLI of 0.903, a RMSEA of 0.041, and a WRMR of 1.260. There was a deterioration of the goodness-of-fit indices when comparing the CFA to EFA, because of the restrictions imposed on the model. Nevertheless, this deterioration was not to the degree that it would severely compromise the acceptability of the fit. The resulting factors, with their corresponding factor indicators and factor loadings, as well as their threshold parameters and item locations, are presented in Table 3. Most factor loadings scored high on their respective latent factors, meaning strong association with their purported depression dimension. Item threshold parameters provide insight in regards to the relative location of the item along the latent factor of depression. Briefly, item-level analysis revealed that the multidimensional depressive construct could be organized into a continuum of severity in the following ascending order: sexual, cognitive, insomnia, appetite, non-interactivenesss/motor retardation, and agitation.

**Table 3:** Confirmatory Factor Analysis with factor loadings and response option thresholds.

Depression dimension	Item content	Factor loading	Response option thresholds				Loc	R2
			B1	B2	B3	B4		
**Insomnia**								
HAM5	insomnia late	0.665	-0.424	0.079			0.172	0.44
HAM6	insomnia middle	0.678	-0.387	0.326	2.803		0.913	0.459
BDIQ16	insomnia	0.709	-1.491	-0.226	0.278		-0,482	0.502
**Non-interactiveness / Motor Retardation**								
CORE17	slowing of speech	0.722	1.046	1.750	2.806		1.865	0.521
HAM8	psychomotor retard.	0.794	0.060	1.102	2.431		1.197	0.630
CORE6	delayed verbal responses	0.764	-0.148	0.559	1.959		0.787	0.583
CORE16	talk impaired spontaneity	0.872	0.849	2.004	2.806		1.884	0.761
CORE7	shortened verbal responses	0.853	1.014	2.107			1.5600	0.727
CORE13	slowed movement	0.522	0.983	2.325			1.654	0.272
CORE12	poverty of associations	0.735	0.626	1.620	2.806		1.682	0.540
CORE2	facial immobility	0.756	0.962	1.844	2.575		1.792	0.571
CORE1	non-interactiveness	0.801	0.515	1.721			1.118	0.641
CORE10	bodily immobility	0.714	1.044				1.044	0.510
CORE15	delayed motor activity	0.862	0.673	1.620	2.575		1.621	0.743
CORE4	non-reactivity	0.883	0.376	1.721	2.806		1.632	0.780
CORE8	inattentiveness	0.885	0.244	1.238	2.325		1.267	0.784
**Agitation**								
CORE9	facial agitation	0.830	0.417	1.404	2.575		1.463	0.689
CORE11	motor agitation	0.782	1.355	2.107	2.806		2.087	0.611
HAM9	agitation	0.876	0.787	1.918			1.352	0.767
CORE5	facial apprehension	0.590	1.844	2.806			2.325	0.348
CORE14	verbal stereotypy	0.751	0.472	1.035	2.053	2.575	1.531	0.564
HAM10	anxiety psychological	0.628	-1.068	0.303	0.914	1.918	0.513	0.395
**Cognitive Symptoms (Pessimism)**								
BDIQ5	feelings of guilt	0.640	-1.811	-0.383	0.544		-0.551	0.409
BDIQ3	feelings of failure	0.541	-1.147	-0.101	0.334		-0.918	0.293
BDIQ7	disappoint in myself	0.727	-0.803	-0.120	0.794		-0.044	0.528
BDIQ6	feelings of punishment	0.414	-1.844	-0.199	0.403		-0.547	0.172
BDIQ8	critical of myself	0.676	-0.618	0.098	0.680		0.053	0.457
BDIQ9	suicidal thoughts	0.574	-0.387	-0.088	0.031		-0.148	0.330
BDIQ12	lost interest in people	0.705	-0.840	0.508	1.102		0.256	0.497
BDIQ13	difficulties in decisions	0.579	-1.293	-0.314	0.513		1.097	0.336
BDIQ1	feelings of sadness	0.701	-0.489	0.511	0.873		0.297	0.491
BDIQ2	hopelesness	0.592	-0.686	0.095	1.123		0.176	0.351
BDIQ4	anhedonia	0.606	-1.532	-0.847	0.624		-0.586	0.368
HAM3	suicide	0.477	-0.801	-0.213	0.750		-0.088	0.228
BDIQ14	unattractiveness (feel.)	0.305	-0.445	0.193	1.575		0.439	0.093
HAM2	feelings of guilt	0.632	-0.641	-0.199	0.762	2.431	0.587	0.400
**Appetite**								
HAM16	weight loss	0.778	-0.412	0.331	1.053		0.323	0.606
BDIQ19	weight loss	0.662	0.355	0.978	1.434		0.921	0.439
HAM12	GI symptoms	0.799	-0.123	1.212	2.806		1.296	0.638
BDIQ18	worse appetite	0.734	0.559	0.822			0.690	0.538
**Sexual**								
HAM14	genital symptoms	0.692	-0.993	-0.465	2.806		0.447	0.478
BDIQ21	loss of libido	0.935	-1.108	-0.369	0.038		-0.482	0.875

Legend: HAM, 17-item Hamilton Depression Rating Scale; BDI, 21-item Beck Depression Inventory; CORE, Core Assessment of Psychomotor Change; Loc, items locations; R2, squared factor loading (proportion of variance in that indicator variable explained by the factor).

## Discussion

The present study was able to identify a six-dimension solution, capturing the multidimensionality of the depressive construct and organizing the items into factors in an ascending order of a continuum of severity, as follows: sexual, cognitive, insomnia, appetite, non-interactiveness/motor retardation, and agitation. Being that depression is a multidimensional construct, it is likely that comprehensive assessments should increase the ability to provide information about it.

Confirmatory Factor Analysis showed that six latent factors were capable of successfully capturing the variance of scale items. An interesting finding was that each dimension was discriminated at different levels of severity (Table 3). It seems that the different symptomatic scales measure different aspects of depression, with the BDI being more linked to the cognitive domain (many items in the cognitive dimension of depression coming from the BDI), and the CORE more connected to the melancholic (psychomotor) domains (Table 3).

A cognitive dimension emerged mostly from a self-reported instrument, and the non-interactiveness/motor-retardation dimension originated from a clinician-rated instrument specifically designed for evaluating psychomotor signs of depression (namely, melancholic depression), while the other four dimensions had a more mixed profile of items from different scales. It was interesting to observe that a self-reported instrument constructed with the objective of measuring responses from patients receiving psychotherapy assessed a profile of more subjective symptoms, in accordance with its conceptual grounds. The same reasoning is true for the CORE measure, as it assesses objective signs of psychomotor disturbance. This set of findings points to the advantages of combining methods in order to have a comprehensive assessment of the depressive phenomena. Whereby, instruments are based on theories of what depression is supposed to be, and as a consequence they inevitably carry the bias of how they were conceptualized. When different scales are integrated, with different theoretical backgrounds, we are tentatively integrating perspectives of depression that are different, yet complementary (and sometimes overlapping).

The study is not without limitations. The analysis was restricted to a single sample and replication of the current model in different populations is needed to confirm the validity of the proposed model. The sample is restricted to outpatients with depression, and the inclusion of both community samples (at the least severe end) and inpatients (at the most severe end of the spectrum) would be important to extend the model to more and less severe manifestations of depression. The study also has some important strengths. The six latent factors uncovered in the study, besides presenting adequate goodness-of-fit indices (*statistically* speaking), are also clinically sound and intuitive, matching fairly closely factor structures hypothesized in previous studies [6]. The importance of such a result is multifold.

*First*, dimensions of depression might represent simpler hints to biological underpinnings than the whole depressive syndrome, which can result in more straightforward, and perhaps successful, etiological investigations (akin to the concept of *endophenotypes*) [32]. Psychiatry has had difficulty in identifying genes responsible for MDD [33], and a probable reason for that is the degree of complexity and heterogeneity of its phenotypic presentation. Therefore, decomposing the depressive phenotype into more elementary structures could result in more straightforward pathways to putative candidate genes. There is evidence that relates specific dimensions of depression to elevated concentrations of proinflammatory cytokines [34]. *Second*, dimensions of depression could allow studies investigating their association to risk factors of depression, such as history of childhood trauma, medical conditions, medications, etc. There is evidence that points to the existence of biologically distinguishable subtypes of depression as a function of childhood trauma [35]. *Third*, dimensions of depression might be tested as predictors of differential treatment outcomes in studies on antidepressant medications. Most studies to date fail in identifying specific clinical predictors for antidepressant treatment, and a possible reason for that is the non-specificity of the diagnosis of MDD, which, unsurprisingly, would lead to non-differential treatment outcomes [36]. In this manner, evaluating depression dimensions could be a way of refining the diagnosis of MDD and increasing the probability of finding better treatment predictors. Uher et al. [37], in a multicenter clinical study, investigated the hypothesis that tricyclic antidepressants and serotonin reuptake inhibitors are equally effective for depression. Mixed-effect linear regression showed no difference between escitalopram and nortriptyline on the three original scales used to evaluate treatment effectiveness: the Montgomery-Asberg Depression Rating Scale, the Hamilton Depression Rating Scale, and the Beck Depression Inventory. However, results based on a model comprising three dimensions (observed mood, cognitive symptoms, and neurovegetative symptoms) from these three scales in combination, revealed drug-specific advantages: observed mood and cognitive symptoms improved more with escitalopram than with nortriptyline, and neurovegetative symptoms improved more with nortriptyline than with escitalopram. These results indicate the possible utility of dimensional symptom measures derived by psychometric analysis from different sources to determine relative advantages of individual antidepressants.

The consequences of the current findings to research and clinical care are: 1) the identification of dimensions of depression based on variables across different units of analysis, what may provide a more comprehensive and detailed assessment of MDD–this is in line with novel approaches, like the RDoC, that aim at an integrative understanding of psychopathology for mental illnesses [38]; and 2) to provide a better heuristic framework for physicians to use while in clinical practice than the current one–that conceives MDD as a “monolithic” entity–, what may allow better-tailored and personalized interventions.

In conclusion, six factor dimensions were extracted from three instruments assessing different aspects of depression, and it is proposed that these factor dimensions could be used in assisting in the refinement of the diagnosis in clinical and research settings, in informing etiological explorations, and in serving as a basis for studies in the pursuit of finding differential treatment predictors.

## Supporting Information

S1 File Exploratory Factor Analyses factors (2–10 factors). (XLS)
